# From religious to existential issues: the implications for GPs: Commentary on: ‘Development of the EMAP tool facilitating existential communication between general practitioners and cancer patients’

**DOI:** 10.1080/13814788.2017.1380792

**Published:** 2017-11-20

**Authors:** Erik Olsman, Dick L. Willems

**Affiliations:** a Section of Medical Ethics, Department of General Practice, Academic Medical Centre, University of Amsterdam, Amsterdam, The Netherlands;; b Section of Ethics and Law of Healthcare, Department of Neurology, Leiden University Medical Centre, Leiden, The Netherlands;; c Department of Spiritual Care, Hospice Bardo, Hoofddorp, The Netherlands

Some weeks ago, one of us (EO) had the privilege to teach senior general practitioners (GPs) who supervise GP trainees, on existential and spiritual care for patients. Teaching this subject matter to GPs at a university that was originally socialist would have been strange, if not impossible, two or three decades ago. By then, spirituality was equated with religion and religion belonged to religious institutions, like churches, and these institutions were associated with power. Supported by the influential philosophers of suspicion—Nietzsche, Freud, and Marx—and by emancipatory movements in the sixties and seventies, most academics thought religion was at its best a hobby of the individual and its worst the opium for the people or an infantile regression.

Why then, were these senior GPs eager to learn about spirituality and existential care? What has changed? The answer is that the backgrounds of religion and spirituality have changed. Twentieth-century sociologists of religion and spirituality found their ‘secularization thesis’ confirmed often. Put simply; this thesis means that religion and everything that belongs to it—practices, beliefs, experiences, et cetera—eventually will disappear. In part, this is true: ask the church leaders. However, in the last two decades, a new thesis has been introduced: the ‘transformation thesis.’ In their groundbreaking work, Heelas and Woodhead suggested that religion was giving way to spirituality: conformity to religious institutions has decreased, whereas individuals increasingly draw from several existential and spiritual sources, like arts, music, stories, etc., nourishing their inner lives and subjective well-being [1]. Some, like the authors of the article on the development of the EMAP tool, prefer the word ‘existential,’ which acknowledges the non-religious elements of spirituality [2]. Others, such as the authors of the 2017 WHO definition of palliative care, speak about ‘spiritual,’ which includes these existential dimensions [3].

Regardless of these terminological issues, we should ask the question: if the transformation thesis is indeed correct, why is this relevant for general practice in European countries? The most important reason is that many people, particularly those facing illness and deterioration, are struggling with existential or spiritual issues but they decreasingly turn to their religious leaders, like priests or ministers, and maybe imams. One consequence may be that they increasingly turn to their GPs to discuss these issues and find existential support. Alternatively, perhaps they do not increasingly turn to their GPs but still turn to their GPs with their existential issues, as they did in earlier decades.

However, GPs may experience a lack of time and training to discuss these matters with their patients [4]. The senior GPs in the classroom expressed a lack of competence to discuss these issues with their patients. One possible explanation is that, compared to a couple of decades ago, people are no longer either religious or non-religious but rather, there are far more than two groups. Plurality is the current state of affairs: a wide variety of existential practices, convictions and experiences. Not only spiritual care providers but also other healthcare providers face this plurality in their daily work [5]. The article by Assing Hvidt et al. offers a helpful tool that may support GPs to discuss existential topics with their patients with a variety of existential backgrounds [2]. Interestingly, the authors stress several times that theirs is a mapping tool (hence the somewhat far-fetched acronym EMAP), intended to chart the patient’s existential problems and resources [2]. The fact that they do not think of it as a diagnostic tool indicates that they rightfully feel that they take us into a nonmedical realm.

That is where some questions remain: are doctors—and GPs specifically—the ones to discuss these issues with their patients? The implicit idea of the paper by Assing Hvidt et al. is that, with the help of this conversation tool, any GP could provide existential support to cancer patients who need this. Maybe that is right: existential questions belong to our shared humanity, and as such, they may not demand specific expertise but structured attention and an empathic attitude. GPs do not need to be experts in existential issues; they primarily need to be open for these problems.

Therefore, we sympathize with the idea that GPs are in a position to at least be open to existential worries and concerns of their patients but we have doubts about the sufficiency of introducing a conversation tool, even if helpful. During the last decades, mainstream general practice, with its focus on the use of evidence, has gone in a direction that, if anything, is opposed to discussing existential issues with patients. Moreover, in most European countries, the system of healthcare financing will hardly encourage GPs to take 30 min for a conversation about spiritual issues that is nowhere to be found in any reimbursement scheme.

It does not mean that GPs will not try to find time to discuss these issues with patients. Neither are they only focused on getting money for this work. Rather, we argue that when we want to support GPs in discussing existential issues with severely ill patients, as the EMAP proposes, a rethinking of primary healthcare issues at a societal level is necessary. For example, one of the issues the senior GPs in our classroom raised was that when they discussed these issues with patients, they faced the lack of expert existential/spiritual care providers in primary care settings to whom they could refer patients with existential problems. Until recently in Dutch palliative care, spiritual care in community settings was not reimbursed, and the reimbursement of spiritual care in other primary care settings than palliative care ones is still under debate.

As the authors say in the last sentence of the discussion, they developed the instrument with more than cancer patients in mind. Even though the existential problems and resources of, for instance, COPD patients may differ from those of cancer patients (and between types of cancer), their tool indeed seems generic enough to be useful in other patient groups.

As good articles do, the article by Assing Hvidt et al. may raise more interesting questions than it solves. The authors deserve praise for opening this important perspective for general practice. We hope that their tool will be helpful for GPs.
